# Supplementary material for: Epstein-Barr Virus DNAemia and post-transplant lymphoproliferative disorder in pediatric solid organ transplant recipients
Source: PLoS One. 2022 Oct 18;17(10):e0269766. doi: 10.1371/journal.pone.0269766 (PMC9578615; doi:10.1371/journal.pone.0269766)
Supplement: S1 Data — (DOCX) [file pone.0269766.s001.docx]

Supplemental Data

Table 7

| Total number of EBV tests 4614 | | | | Median number of tests per patient 12 | | | | |
| --- | --- | --- | --- | --- | --- | --- | --- | --- |
| Type of organ transplant | | Total number of subjects | Missing | Deceased < 1 yr post-transplant |  | Median number of tests for all patients | Median number of tests for subjects EBV+ by PCR | Median number of tests for subjects EBV- by PCR |
|  |  |  |  |  |  |  |  |  |
|  | Heart | 73 | 5 | 5 |  | 11 | 12.5 | 7 |
|  | Intestine | 10 | 1 | 0 |  | 43 | 50 | 27 |
|  | Kidney | 38 | 1 | 0 |  | 10 | 10.5 | 10 |
|  | Liver | 134 | 5 | 1 |  | 14 | 19 | 8 |
|  | Lung | 19 | 8 | 1 |  | 1.5 | 3 | 1 |
|  | Multivisceral | 20 | 0 | 0 |  | 18.5 | 34 | 15 |

Table 8

| Median number of tests done per subject, stratified by year and only including tests done within first year post-transplant | | | | | | | | |
| --- | --- | --- | --- | --- | --- | --- | --- | --- |
| Year | Total including deceased | Total excluding deceased |  | EBV- by PCR | EBV+ by PCR |  | Liver transplant only | Non-liver transplants |
| 2007 | 6 | 7.5 |  | 2 | 9 |  | 16 | 2 |
| 2008 | 6 | 6 |  | 0 | 6 |  | 10.5 | 4 |
| 2009 | 5 | 5 |  | 4 | 5 |  | 9 | 2 |
| 2010 | 7 | 8 |  | 6 | 8 |  | 10.5 | 2.5 |
| 2011 | 8 | 8 |  | 9 | 8 |  | 12.5 | 4.5 |
| 2012 | 7 | 7 |  | 6 | 7.5 |  | 11 | 4 |
| 2013 | 11 | 11 |  | 10 | 13 |  | 13 | 10 |
| 2014 | 8.5 | 9 |  | 7 | 11 |  | 11 | 9 |
| 2015 | 8 | 8 |  | 7.5 | 8.5 |  | 9.5 | 7 |
| 2016 | 7 | 7 |  | 6.5 | 8 |  | 6.5 | 10 |
| 2017 | 6 | 6.5 |  | 5 | 9 |  | 6 | 11 |
| 2018 | 8 | 8 |  | 7 | 9.5 |  | 6 | 12 |
