# Supplementary material for: Epstein-Barr Virus DNAemia and post-transplant lymphoproliferative disorder in pediatric solid organ transplant recipients
Source: PLoS One. 2022 Oct 18;17(10):e0269766. doi: 10.1371/journal.pone.0269766 (PMC9578615; doi:10.1371/journal.pone.0269766)
Supplement: Attachment — Submitted filename: Response to Reviewers.docx [file pone.0269766.s004.docx]

PLOS One Editorial Board

To Whom It May Concern:

Thank you for the opportunity to resubmit our paper submission number PONE-D-22-15480, titled “Epstein-Barr Virus DNAemia and Post-Transplant Lymphoproliferative Disorder in Pediatric Solid Organ Transplant Recipients”. We have made the following revisions and agree that this has resulted in a much improved paper. We have responded the following points made by the editors:

1. We have edited the section headings to conform with PLOS One’s style requirements
2. Our study was given a waiver for informed consent by our center’s institutional review board (IRB) and this statement has been added to the Materials and Methods. We also added a statement regarding whether data was anonymized before it was accessed and how it was stored.
3. The ORCID iD has been linked and authorized for the corresponding author
4. The ethics statement has been added to the Materials and Methods section
5. We have changed one of the affiliations of the authors per their request. Sonya Kirmani’s affiliation was changed from Duke University, Department of Pediatrics, Division of Pediatric Cardiology to University of Wisconsin-Madison in Madison, Wisconsin, USA.

We have also responded to the following points made by the reviewers.

Reviewer #1:

1. In line 52, the term “organ transplant” has been changed to “type of organ transplant” to correspond to similar language in line 56. We have also changed the headings in all of our tables from “Type of Transplant” to “Type of Organ Transplant” in order to stay consistent.
2. Methods, line 93-94: We did identify diagnosis codes for lymphoma in addition to PTLD and thus, this has been added to the manuscript. As for the comment that there was not an ICD diagnosis code for PTLD, there was one from 2008 forward, and thus, we went 1 year previous to this and started our study in 2007. We did not look at a cancer registry as we did not have access to one and we did not look at problem lists as they are notoriously hard to pull from the charts and also come with their shortcomings. We do realize that there are limitations of identifying PTLD and lymphoma by diagnosis code, and have added this to the discussion section. In the end, we wanted to make sure that the cases of PTLD were indeed true cases of PTLD, proven by biopsy.
3. Methods, line 96: We did use whole blood quantitative EBV PCRs, which as one reviewer mentions is more sensitive but less specific. Indeed, there is much intervariability of quantitative PCRs, and we have included the reference mentioned. In the end, our data is presented not as a specific cutoff but as an association between serial levels using the same laboratory and this may be more universally applicable. This has all been added to the Discussion section as a limitation of this study.
4. Methods, line 100-102: We used only quantitative EBV PCRs from our electronic health system which only pulled in values done at our central laboratory. In checking with the laboratory, they used the same machine over the time period of the study. We also confirmed with them the exact conversion factor, 1 copy/µL = 113.6 IU/mL which has not changed over the study period. All this was added into the manuscript under Methods.
5. We have taken out language regarding logistic regression for PTLD in order to clarify the methods, especially as we do not show these results. Logistic regression was used for the odds of EBV DNAemia, and this is detailed further. In response to how a logistic regression was OK for EBV DNAemia despite the fact that patients develop EBV DNAemia over time, we did notice that a pattern that those transplants who were EBV donor positive/recipient positive (D+/R+) and donor positive/recipient negative (D+/R-) were more likely to develop EBV DNAemia. There were also increased odds of EBV DNAemia for donor negative/recipient positive (D-/R+) subjects although this was not significant. In all of these patients, we noticed that the EBV DNAemia occurred pretty quickly after transplant, and thus, the time to EBV DNAemia was not as variable. As shown in Table 4, not many variables were significantly associated with odds of EBV DNAemia, and the ones that were significant were not time dependent.
6. Results, line 128-129: We take into account the excellent point made on our patient population. We have added the observation that our sample population is heavy in liver transplant recipients, and has less kidney transplant recipients than the typical pediatric center in the Discussion section.
7. EBV positive clarification: We added PCR positive to clarify our definition of EBV positive in Table 1. As further clarification, we also added EBV positive by quantitative PCR to the description “All patients who developed PTLD were EBV positive” on line 136.
8. The reviewer states that in the literature, often D+/R- is one of the highest risk factors for PTLD, and this was not the case in our study. We have added it to the limitations section of this study. We are not sure why our data does not match this, it could be a sample size issue, something intrinsic to our population, or the fact that providers much more carefully followed patients if they were D+/R-.
9. Results line 155: The odds ratios for EBV D+/R- and D+/R+ were indeed switched and we have edited this to correctly correspond with the proper EBV serostatus category.
10. We have added more in the Discussion section as to why there are differences between risk factors for PTLD (Table 2), and risk factors for time to PTLD (Table 6). There is an additional variable in the analysis in Table 6, the log­_10_ transformed maximum level of EBV. Also, in the analysis in Table 6, each variable is adjusted against the others. Lastly, with the analysis of time to PTLD, we do realize that we cannot study all the variables that vary over time such as immunosuppression and this may confound the results as well. I think it is interesting to see that Table 2 presents the systematic overview of associations and is a good frame of reference, and Table 6 presents variables that may be more important for individualistic decision-making.
11. While we do have granular EBV quantitative levels, there are too many changes in the patients’ immunosuppressive maintenance regimens over time and we could not study changes in all the patients. While we can pull out T cell depleting agents such as anti-thymocyte globulin (ATG) and alemtuzumab (Campath), it is very hard to categorize all the small changes to maintenance immunosuppression. In our center, changes to medications could reflect a change in levels or an actual adjustment in immunosuppression level. This would require a massive chart review as clinical notes would be the gold standard here, in order to generate a detailed summary of immunosuppression changes, which would be extremely time intensive. Patients do obtain troughs but these values are also variable and do not always reflect true goals. We would have to do a chart review looking at clinical notes and as patient immunosuppressive levels change over time, it would be hard to categorize changes. We do state this as a major limitation of this study.

Reviewer #2:

1. We understand the issue of testing by indication bias, and have added this to the limitations. This is a very important point. We have additional tables in the Supplementary Data section that does confirm this. Whether stratified by year or type of organ transplant, for the most part, those patients who are EBV positive by PCR have a higher median number of tests when compared to those who are EBV negative PCR. Per our protocol and guidelines, those who have higher levels of EBV DNAemia are monitored more closely and yes, there is a lower threshold to decrease immunosuppression in these patients. However, to the point of sudden EBV DNAemia (primary infection) resulting in PTLD, as you can see from Figure 1, the vast majority of patients who developed PTLD had high EBV levels for about 2 months or so before developing PTLD. There weren’t a lot of patients where the development of PTLD was caught by surprise. We do realize that a better summary of how EBV testing was done can help frame our reference, and we have thus included this extra data in the Supplemental Data section.
2. We have added the specific comment that teams could decrease immunosuppression as a response to high EBV levels and thus reduce risk for PTLD to our Discussion section. There is no easy way to delineate all the changes in maintenance immunosuppression at our center and as previously mentioned, we have stated this as a major limitation.
3. Lung transplants did not have much EBV DNAemia but had high hazards ratio for PTLD. This is likely due to testing nuances at our center. Lung transplants are followed for only a short time period at our center, and most of them receive EBV levels as external labs which we cannot capture easily. We do have a biased sample of this population because those who stay at our center often are those who are sicker, gone through rejection, and of course, they stay or become referred back into our care if they are diagnosed with PTLD.
4. We did not see more EBV over time. We do have some raw data on this. What we did see was more frequent monitoring at our center in the heart and kidney transplant recipients, which likely led to immunosuppression adjustments over time. Multivisceral/intestinal transplant recipient EBV monitoring was kept constant, and monitoring in liver transplant recipients actually decreased over time, but we did not see as much PTLD in liver transplant recipients. Please see the Supplemental Data section for this.

Minor Comments:

Table 1: In order to clarify the phrase “EBV positive post-transplant”, we added the words “PCR positive” was added

Line 136: To clarify the phrase “all cases of PTLD were EBV positive”, this was edited to refer to PCR positivity prior to PTLD diagnosis.

Line 138: For the comment on confidence intervals, it is very hard to put a confidence interval on time to PTLD, the curve is non-parametric. Also, with the hazard ratios that had smaller sample size, the confidence interval may be extremely wide and harder to interpret.

Table 6: The 1957 on the CI of the hazards ratio for lung transplants is not an error. Due to the very limited sample size of lung transplants (we only had one positive PTLD case amongst 19 cases, and if you looked at the EBV data for lung transplants, it was very sparse as most are referred back to a closer home institution after transplant for EBV monitoring there.

We have responded to all the comments by the reviewers and look forward to your response.

Sincerely,

Yeh-Chung Chang, MD

Assistant Professor of Pediatrics

Duke University School of Medicine

Department of Pediatrics, Division of Infectious Diseases

315 Trent Drive, Room 371

Durham, NC 27705

dan.chang@duke.edu
